# Arterial Stiffness: Effects of Anticancer Drugs Used for Breast Cancer Women

**DOI:** 10.3389/fphys.2021.661464

**Published:** 2021-05-13

**Authors:** Giuseppina Novo, Daniela Di Lisi, Roberta Manganaro, Girolamo Manno, Simone Lazzara, Federico Angelo Immordino, Cristina Madaudo, Scipione Carerj, Antonio Russo, Lorena Incorvaia, Concetta Zito

**Affiliations:** ^1^Department of Health Promotion, Mother and Child Care, Internal Medicine and Medical Specialties, University of Palermo, Cardiology Unit, University Hospital P. Giaccone, Palermo, Italy; ^2^Department of Clinical and Experimental Medicine, Section of Cardiology, University of Messina, University Hospital “Policlinico G. Martino”, Messina, Italy; ^3^Department of Surgical, Oncological and Oral Sciences, Section of Medical Oncology, University of Palermo, Palermo, Italy; ^4^Department of Biomedicine, Neuroscience and Advanced Diagnostics (Bi.N.D.), Section of Medical Oncology, University of Palermo, Palermo, Italy

**Keywords:** arterial stiffness, vascular injury, pulse wave velocity, cardiotoxicity, chemotherapy

## Abstract

**Purpose:** It is well known that anticancer drugs used for treating breast cancer can cause cardiac toxicity, and less is known about vascular toxicity. The aim of this study was to assess subclinical vascular effects of anthracyclines and trastuzumab (TRZ) in women treated for breast cancer.

**Methods:** We enrolled 133 female patients with breast cancer undergoing adjuvant treatment with anthracycline-containing chemotherapy (CT) followed by taxane (paclitaxel/docetaxel) + TRZ. Patients underwent a standard echocardiography including measurement of left ventricular ejection fraction and global longitudinal strain at baseline and at follow-up. Vascular toxicity was evaluated by measuring brachial blood pressure (BP) and arterial stiffness indices (pulse wave velocity and Beta stiffness index) at T0 (baseline), T1 (3 months), T2 (6 months), and T3 (12 months).

**Results:** Arterial stiffness indices were significantly increased at T1 in patients treated with anthracycline-containing CT (PWV 5.5 m/s IQR 5.15–6.4 at T0 vs. PWV 6.7 m/s IQR 5.6–7.2 at T1, *p* < 0.05; Beta index PWV 6.7 IQR 5.25–6.65 at T0, PWV 8.35 IQR 6.5–10.15 at T1, *p* < 0.05) but not at T2 and T3, when treatment with anthracyclines was stopped and patients were under treatment with taxane and TRZ. Blood pressure values did not significantly change during follow-up.

**Conclusion:** Changes in arterial stiffness parameters occur early after starting treatment with anthracyclines, and they seem to be reversible if anthracycline treatment is stopped. These changes are not influenced by blood pressure values modifications. Therefore, in breast cancer women, anthracyclines seem to cause early reversible subclinical vascular injury.

## Introduction

Cardiotoxic effects of anthracyclines and their mechanism have been extensively studied (Zhang et al., 2012). It is known that left ventricular dysfunction and heart failure are the main toxic effects of anthracyclines (Swain et al., 2003). Recently, some studies revealed structural arterial remodeling, increased arterial stiffness, and endothelial dysfunction after anthracyclines therapy in patients with hematologic malignancies (Mozos et al., 2017). A significant increase in aortic stiffness was found very soon, within 4 months of anthracycline therapy initiation, in a study including patients with lymphoma or leukemia, regardless of age, sex, diabetes, hypertension, hyperlipidemia, or coadministration of other cytostatics. The rapid increase in pulse wave velocity (PWV) at 1 month suggested an acute process (endothelial dysfunction, increase in smooth muscle tone) rather than a chronic one (atherosclerosis, increased collagen synthesis; Drafts et al., 2013). Daskalaki et al. studied 70 patients with lymphomas who underwent therapy with anthracyclines, reporting a progressive, dose-dependent decrease in aortic distensibility (Daskalaki et al., 2014). In another study, also in survivors of childhood cancer, who completed anthracyclines therapy for a malignant disorder, aortic PWV was significantly higher compared to age- and sex-matched healthy controls (Herceg-Cavrak et al., 2011). In breast cancer women, after chemotherapy, arterial stiffness resulted higher compared to controls (Yersal et al., 2018). Thus, anthracyclines may influence aortic stiffness through several mechanisms and arterial stiffness could be used as a predictive marker of subclinical vascular damage. Arterial stiffness has been shown to be an independent risk factor for future cardiovascular diseases (Novo et al., 2010). The aim of this study was to assess vascular toxicity induced by anthracyclines and other anticancer drugs in breast cancer women, measuring arterial stiffness. In addition, we assessed myocardial deformation indices at echocardiogram to detect an early cardiac dysfunction.

## Materials and Methods

After their informed consent was collected, 133 consecutive female patients with diagnosis of breast cancer were enrolled. The study was approved by the Ethics Committee of the University Hospital P. Giaccone in Palermo. Inclusion criteria were new diagnosis of breast cancer, basal left ventricular ejection fraction (LVEF) >50%, absence of coronary artery disease and hemodynamically significant valvular heart disease, absence of carotid atherosclerotic plaques, absence of significant comorbidities, and adequate echocardiographic imaging before starting anthracycline-containing CT in adjuvant setting. Exclusion criteria were age under 18, preexisting left ventricular dysfunction before starting chemotherapy, and severe liver and renal dysfunction. Careful clinical history was collected, and the presence of cardiovascular risk factors was evaluated (diabetes mellitus, dyslipidemia, smoke habit, and arterial hypertension). All patients underwent blood pressure measurement, electrocardiogram with QTc interval measurement, standard transthoracic echocardiogram, and carotid ultrasound scan with arterial stiffness measurement at baseline before starting CT (T0), at 3 (T1), 6 (T2), and 12 (T3) months. The CT protocol included anthracycline for four cycles with an inter-cycle interval of 21 days (3 months-T1). After anthracycline-based CT, patients continued treatment with taxane (paclitaxel/docetaxel; 57%), trastuzumab (TRZ; 31%), radiotherapy (51%), and endocrine therapy (37%).

Standard echocardiogram was performed using a Vivid E9 ultrasound machine (GE Healthcare) equipped with a 2.5 MHz transducer and included speckle tracking analysis (AFI, GE). Chamber and ventricular function quantifications were measured according to the ASE/EACVI guidelines (Lang et al., 2015). Diastolic function was evaluated measuring the trans-mitral Doppler flow and the tissue Doppler imaging at septal and lateral mitral annulus, indexed left atrial volume, and tricuspid regurgitation peak velocity (Nagueh et al., 2016). Global longitudinal strain (GLS) of left ventricle was measured off-line on 2D images of three consecutive cardiac cycles from the three apical views at an approximately equal heart rate. GLS was considered in absolute value according to chamber quantification recommendations (Lang et al., 2015). Cardiotoxicity was defined as a variation of EF >10% below 50%. A relative GLS reduction ≥15% at follow-up compared to baseline was defined subclinical cardiotoxicity (Zamorano et al., 2016).

Carotid arteries ultrasound scan was performed using an Esaote machine (MyLab Twice) with a high-resolution 12 MHz linear sequence transducer. The device was equipped with RF Quality Intima-Media Thickness Analysis and Radiofrequency (RF) Quality Arterial Stiffness (QAS) analysis software. This software measures arterial stiffness at a single region of interest (on the common carotid artery, 1 cm below the carotid bifurcation), by monitoring arterial wall movements, by RF signals, in systolic and diastolic phases during six cardiac cycles, in B mode examinations, and it is able to detect micrometric variations of the artery diameter. The arterial distension waveform is combined with the brachial artery blood pressure (BP), which was measured after approximately 10 min of resting, before starting the examination and recorded in the device. Among the detectable parameters, we reported PWV and parameter *β*. PWV is the travel speed of the pulsed wave and is expressed in meters per second. The parameter *β* indicates the degree of atherosclerosis, and it increases in the presence of atherosclerosis.

Age- and sex-specific percentiles of local carotid stiffness in healthy population were reported in the literature.

## Statistical Analysis

Quantitative variables are reported as mean and standard deviation (SD) or median and interquartile range (IQR) according to data distribution analysis as verified by D’Agostino–Pearson test; qualitative variables were reported as percentage. ANOVA was performed using Friedman test. Multiple regression analysis has been used to evaluate the influence between variables. Differences were considered significantly if *p* < 0.05. All analyses were performed using the MedCalc Statistical Software version 19.2.1 (MedCalc Software Ltd., Ostend, Belgium; https://www.medcalc.org; 2020).

## Results

**Figure 1:**
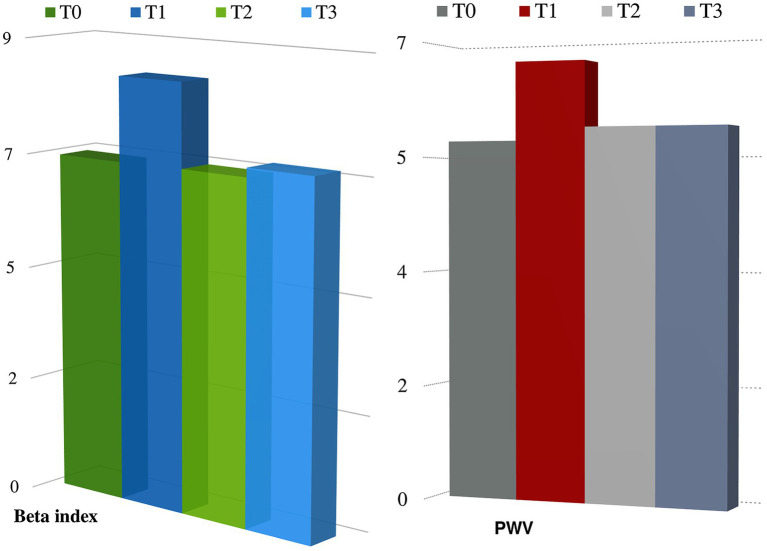
Changes in arterial stiffness parameters during follow-up. *p* < 0.05 at T1 compared to T0 either for PWV and beta index.

Table 1 summarizes the characteristic of the study population (133 patients, mean age 55.64 ± 11.74 years). In the overall population, 21.8% of patients were hypertensive, 13.5% were affected by diabetes mellitus, 21.8% were affected by dyslipidemia, 18% had a family history of cardiovascular diseases, and 13.5% were smokers. During the follow-up, after chemotherapy, 4.5% patients had new onset arterial hypertension and 25% reported palpitations. None had myocardial infarction or angina pectoris, and effort dyspnea was observed in five patients. We did not find significant changes in QTc interval during the follow-up, no significant ventricular and supraventricular arrhythmias were observed. Analyzing arterial stiffness, we found a significant increase at T1, as well as a decrease with a return to basal values at T2 and T3. Particularly, median values of Beta index increased significantly at T1 (T0: 6.7 IQR 5.25–6.65 vs. T1: 8.35 IQR 6.5–1.15, *p <* 0.05), but there were no any significant variations at T2 (6.72 IQR 5.5–8.1) and T3 (6.9 IQR 5.75–9) compared to baseline. We also found a statistically significant increase in PWV at T1 (T0: 5.5 m/s IQR 5.15–6.4 vs. T1: 6.7 m/s IQR 5.6–7.2, *p* < 0.05); accordingly, variation was not significant at T2 (5.75 m/s IQR 5.2–6.7) and T3 (5.7 IQR 5.15–6.6; Figure 1). Arterial blood pressure is not significantly modified between times (see Table 2). At multivariate regression analysis including median blood pressure values, pulse pressure, and Beta index, only Beta index was independently related to PWV (Beta index: T0 *p* < 0.0001; T1 *p* < 0.0001; T2 *p* < 0.0001; and T3 *p* < 0.007), while pressures lost predictivity at T1 (*p* > 0.05).

**Table 1:** General characteristics of the study population.

	Mean ± SD/median (IQR)/*n* (%)
Population (*n*)	133
Age (years)	55.64 ± 11.74
Height (cm)	160 ± 6.8
Weight (kg)	68 (58–78)
BSA (mq)	1.7 (1.6–1.83)
BMI (kg/mq)	26.4 (22.1–29.9)
Family history of CVD	24 (18%)
Diabetes	18 (13.5%)
Hypertension	292 (1.8%)
Dyslipidemia	29 (21.8%)
Smoking	18 (13.5%)
ACE-I	11 (8.27%)
Sartans	8 (6%)
B-blockers	11 (8.27%)
Diuretics	9 (6.7%)

BMI, body mass index; CVD, cardiovascular disease; ACE-I, angiotensin-converting enzyme inhibitors; B-blockers, beta-blockers.

**Table 2:** Comparison of various parameters at different time of evaluation with Friedman test (value of *p* refers to the changes that occur in the various times compared to the baseline).

Value	T0	T1	T2	T3
PWV (m/s)	5.5 (IQR 5.15–6.4)	6.7 (IQR 5.6–7.2) *p* > 0.004	5.75 (IQR 5.2–6.7) *p* > 0.05	5.7 (IQR 5.15–6.6) *p* > 0.05
Beta	6.7 (IQR 5.25–6.65)	8.34 (IQR 6.5–10.15) *p* > 0.01	6.72 (IQR 5.5–8.1) *p* > 0.05	6.9 (IQR 5.75–9) *p* > 0.05
SBP (mmHg)	120 (IQR 110–140)	130 (IQR 115–135) *p* > 0.05	130 (IQR 120–140) *p* > 0.05	130 (IQR 120–140) *p* > 0.05
DBP (mmHg)	80 (IQR 70–80)	80 (IQR 70–87.5) *p* > 0.05	80 (IQR 70–80) *p* > 0.05	80 (IQR 75–87.5) *p* > 0.05
MBP (mmHg)	93 (IQR 88.5–100)	93 (IQR 84–103) *p* > 0.05	93 (IQR 88.5–98.5) *p* > 0.05	93 (IQR 89–104) *p* > 0.05
PP (mmHg)	45 (IQR 40–50)	45 (IQR 40–50) *p* > 0.05	45 (IQR 40–50) *p* > 0.05	50 (IQR 40–60) *p* > 0.05
HR (beat/min)	73.5 (IQR 69–97)	72.5 (IQR 71–88) *p* > 0.05	77 (IQR 65–79) *p* > 0.05	77.5 (IQR 62–80) *p* > 0.05
QTc (m/s)	420 (IQR 418.5–429.7)	436 (IQR 407.5–446.5) *p* > 0.05	421 (IQR 429.5–431.7) *p* > 0.05	434 (IQR 405.5–437) *p* > 0.05

SBP, systolic blood pressure; DBP, diastolic blood pressure; MBP, medium blood pressure; HR, heart rate; PWV, pulse wave velocity; Beta, β stiffness index; GLS, global longitudinal strain; EF, ejection fraction; QTc, QT correct.

**Figure 2:**
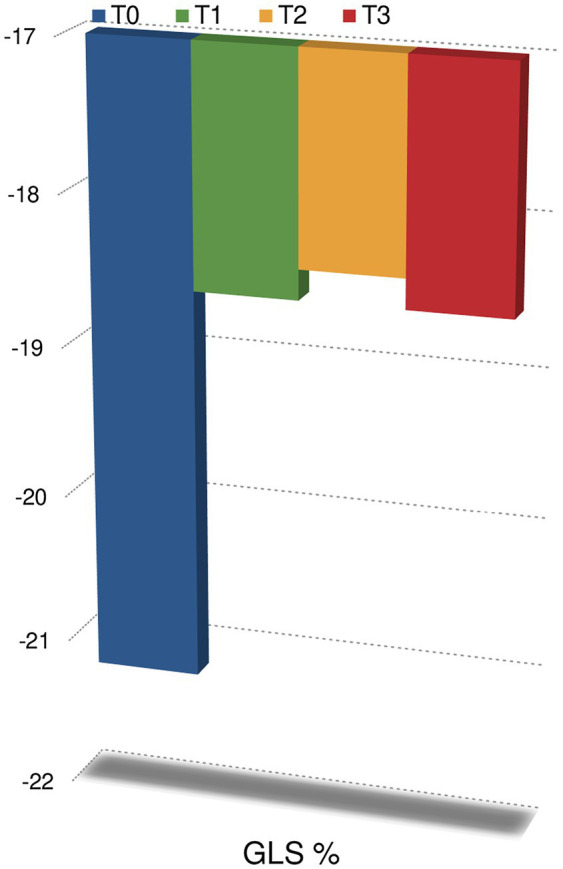
Changes in global longitudinal strain during follow-up. *p* < 0.05 at anytime compared to T0.

Regarding the echocardiographic evaluation, no statistically significant changes in LVEF were observed at T1 (60% IQR 58.2–64.2) compared to baseline (62% IQR 59.7–66, *p* < 0.05); at T2 (59% IQR 55–61) and T3 (58% IQR 56–60), we observed a significant reduction in LVEF compared to baseline (*p* < 0.05). A significant reduction in GLS was observed at T1 (−18.6% IQR −20.8; −17% at T1 vs. −21.2% IQR −22.7; −19.8 at T0, *p* < 0.05), T2 (−18.4% IQR −20; −17.3) and T3 (−18.6% IQR −20.5; −16) compared to baseline (*p* < 0.05; Figure 2). GLS did not significantly changed at T2 and at T3 compared to T1 (*p* > 0.05). Thus, GLS significantly reduced at T1 and persistently reduced at T2 and T3.

None of the patients developed diastolic dysfunction during follow-up nor right ventricular dysfunction (data are reported in Table 3).

**Table 3:** Multiple comparisons of echocardiographic parameters at different time of evaluation according to Friedman test (value of *p* refers to the changes that occur in the various times compared to the baseline).

Value	T0	T1	T2	T3
EDV (ml)	73 (IQR 66–90)	80 (IQR 72–101) *p* > 0.05	81.5 (IQR 67–97) *p* > 0.05	77 (IQR 68–86) *p* > 0.05
LAV (ml)	53 (IQR 44–69)	54 (IQR 43–63) *p* > 0.05	50 (IQR 40.5–63.5) *p* > 0.05	53 (IQR 38–60.5) *p* > 0.05
LVEF (%)	62 (IQR 59.7–66)	60 (IQR 58.2–64.2) *p* > 0.05	59 (IQR 55–61) *p* < 0.05	58 (IQR 56–60) *p* < 0.05
E/E’	7.9 (IQR 6.3–9)	9 (IQR 7.9–10) *p* > 0.05	9 (IQR 7–11.2) *p* > 0.05	8.5 (IQR 7.5–11.47) *p* > 0.05
TAPSE (mm)	22 (IQR 20.25–25.5)	23 (IQR 21–25.75) *p* > 0.05	21 (IQR 19–23) *p* > 0.05	23 (IQR 21–25) *p* > 0.05
GLS (%)	−21.2 (IQR −22.7; −19.8)	−18.6 (IQR −20.8; −17) *p* < 0.05	−18.4 (IQR −20; −17.3) *p* < 0.05	−18.6 (IQR −20.5; −16) *p* < 0.05

EDV, end diastolic volume; LVEF, ejection fraction; LAV, left atrial volume; TAPSE, tricuspid annular plane systolic excursion; GLS, global longitudinal strain.

## Discussion

It is well known that anthracycline-based treatment regimen may induce myocardial damage that is dose-related and can also lead to symptomatic heart failure (Curigliano et al., 2012). The more precocious is the detection of damage and the start of cardioprotective therapy the more reversible it is (Cardinale et al., 2015). Echocardiography is the tool of choice to monitor LV ejection fraction during cardiotoxic cancer treatment. Measurement of GLS that reveals damage of subendocardial fibers is an even more sensitive method to detect subclinical cardiotoxicity (Plana et al., 2018). Patients who receive anthracyclines also exhibit vascular damage; however, the entity of this problem is less clear. Probably, anthracyclines act, as well as cardiovascular risk factors, leading to damage of the subendocardial LV layers along with the endothelial layer of large arteries. This damage can be detected, respectively, by GLS and arterial stiffness measurement. While according to the literature myocardial function deteriorates early and remains abnormal after initiation of anthracyclines regimen if cardioprotection is not promptly started, vascular damage is also acute but it is reversible after stopping oncological treatment.

Our study demonstrates that in breast cancer women treated with anthracycline-containing CT and subsequently taxane and trastuzumab, subclinical vascular damage occurs in the first months of therapy (during administration of anthracyclines) and it seems to be reversible.

Subclinical cardiac dysfunction occurs early and persists over time. In addition, as already known, the reduction in GLS precedes the reduction in LVEF during chemotherapy.

Vascular damage was identified by a significant increase in arterial stiffness parameters (PWV and *β* parameter) at T1; then, it improved at T2 and T3. Our data are in agreement with the few studies present in the literature to date (Chaosuwannakit et al., 2010; Moreo et al., 2016).

Anthracyclines vascular damage is likely to be a direct and acute process due to endothelial dysfunction and increased vascular muscle tone rather than a chronic process due to atherosclerosis and increased collagen synthesis.

Arterial stiffness may be influenced by anthracyclines with different mechanisms. Oxidative stress with the release of free oxygen radicals (induced by anthracyclines; Di Lisi et al., 2017; Novo et al., 2020) would increase arterial stiffness through structural changes within the vascular matrix, interfering with the endothelial regulation of vascular smooth muscle cell tone.

Anthracycline-induced endothelial vascular damage also reduces nitric oxide synthesis and further promotes endothelial dysfunction which in turn increases arterial stiffness. Therefore, reactive nitrogen species act together with reactive oxygen species to damage endothelial cells, causing nitrosative stress Anthracyclines also promote the overexpression of proinflammatory cytokines that can cause endothelial damage (Duquaine et al., 2003). According to this hypothesis in our study, the significant increase in arterial stiffness was observed at T1, in the course of anthracycline therapy, while in the following times, when the patients had stopped this treatment or underwent taxane and TRZ and/or endocrine therapy, it was no longer detected showing a functional and reversible recovery. It is worth of notice that the modification of arterial stiffness was not a consequence of blood pressure increase because it was monitored at each visit and it did not significantly change.

It is known that decreased arterial distensibility can lead to enhanced afterload and subsequent subclinical decline in LV function, as a consequence of ventricular–arterial coupling.

Accordingly, our findings of increased arterial stiffness and reduced GLS at T1 could be interpreted as expression of an early form of abnormal left ventricular–arterial coupling.

However, while GLS reduction starts at T1 and persists till T3, arterial stiffness increases only at T1 with an early recovery at T2. Thus, probably, these findings should be considered separately, as the independent results of cardiac (more persistent) and vascular (acute and early reversible) anthracyclines toxicity, respectively.

Pulse wave analysis is a reliable, noninvasive, cheap, and practical technique. A limitation of our study is the lack of validated standard measurement of arterial stiffness such as carotid–femoral PWV. This is a limitation but also the novelty of our study because single-point measurement of arterial stiffness by QAS has not been used in previous studies. This technique is much more feasible in the clinical context and less time consuming compared to tonometer. It can be performed with the same machine used for echocardiographic examination, and it is only needed to switch the cardiac probe with the linear one and to set the specific software.

## Conclusion

Several studies have demonstrated that arterial stiffness is predictive of vascular events (Laurent et al., 2001); however, it is unclear if in the setting of cancer patients treated with antineoplastic treatments the detection of a transient impairment of vascular function can be a prognostic predictor and further and wider studies with longer follow-up are needed to verify this hypothesis.

Thus, in conclusion, our study confirms that stiffness indexes change early during treatment with anthracycline and this is not a consequence of modifications in blood pressure values. Vascular damage in course of anthracyclines treatment is an acute and reversible damage.

## Data Availability Statement

The datasets presented in this article are not readily available. Requests to access the datasets should be directed to Giuseppina Novo (giuseppina.novo@unipa.it).

## Ethics Statement

The studies involving human participants were reviewed and approved by A.O.U. Policlinico Paolo Giaccone. Informed consent was obtained from all individual participants included in the study.

## Author Contributions

All authors contributed to the writing. GM submitted the manuscript. GN, DL, AR, LI, and CZ are the coordinators of the study. FI and CM analyzed the statistics. LI and CZ are co-last authors. All authors contributed to the article and approved the submitted version.

### Conflict of Interest

The authors declare that the research was conducted in the absence of any commercial or financial relationships that could be construed as a potential conflict of interest.
